# Autophagy in herpesvirus immune control and immune escape

**DOI:** 10.1186/2042-4280-2-2

**Published:** 2011-01-05

**Authors:** Graham S Taylor, Josef Mautner, Christian Münz

**Affiliations:** 1 School of Cancer Sciences and Medical Research Council Centre for Immune Regulation, University of Birmingham, Birmingham, UK; 2 Department of Pediatrics, Technische Universität München and Helmholtz Zentrum München, German Research Center for Environmental Health, Munich, Germany; 3 Viral Immunobiology, Institute of Experimental Immunology, University Hospital Zürich, CH-8057 Zürich, Switzerland

## Abstract

Autophagy delivers cytoplasmic constituents for lysosomal degradation, and thereby facilitates pathogen degradation and pathogen fragment loading onto MHC molecules for antigen presentation to T cells. Herpesviruses have been used to demonstrate these novel functions of autophagy, which previously has been primarily appreciated for its pro-survival role during starvation. In this review, we summarize recent findings how macroautophagy restricts herpesvirus infections directly, how macroautophagy and chaperone mediated autophagy contribute to herpesviral antigen presentation on MHC molecules, and which mechanisms herpesviruses have developed to interfere with these pathways. These studies suggest that herpesviruses significantly modulate autophagy to escape from its functions in innate and adaptive immunity.

## Introduction

Mammalian cells use primarily two proteolytic systems to catabolise intra- and extracellular material for energy and macromolecular building block generation. These are proteasomes and lysosomes. While proteasomes degrade soluble ubiquitinated proteins, lysosomes destroy ubiquitinated protein aggregates and cell organelles. Interestingly, these degradation mechanisms can also be used to eliminate pathogens and process their fragments for presentation to the immune system [1]. Access of substrates to these proteolytic, and in the case of lysosomes generally hydrolytic, machineries is tightly regulated by the 19 s cap complex for the proteasome, and endocytosis, vesicular sorting and autophagy for lysosomes.

**Figure 1:**
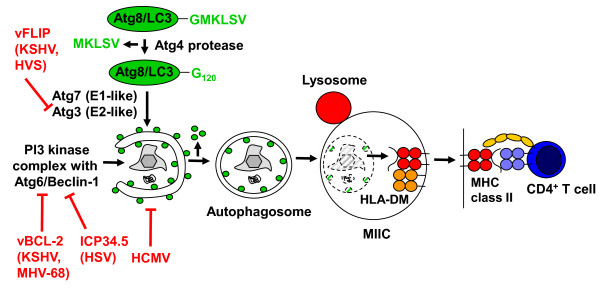
**Immune escape from innate restriction and viral antigen presentation of herpesviruses by macroautophagy**. α-, β- and γ-herpesviruses (herpes simplex virus [HSV], human cytomegalovirus [HCMV], Kaposi sarcoma associated herpesvirus [KSHV], herpesvirus saimiri [HVS] and murine herpesvirus 68 [MHV-68]) inhibit macroautophagy by targeting either Atg6/Beclin-1 to prevent formation of the PI3 kinase complex, marking membranes with PI3 for the assembly of the autophagosome forming machinery, or Atg3, which mediates conjugation of the ubiquitin-like molecule Atg8/LC3, involved in autophagosomal membrane elongation and substrate recruitment. Incompleteness of these inhibitory mechanisms allows for viral particle degradation via lysosomal hydrolysis and antigen presentation on MHC class II molecules to CD4^+ ^T cells.

In this context, autophagy delivers cytoplasmic constituents into lysosomes. Three autophagic pathways have been identified [2]. The first pathway, microautophagy, is a process in which substrates bud into the lysosomal lumen for degradation, but has thus far not been described in higher eukaryotes. The second pathway, chaperone mediated autophagy (CMA), transports proteins that contain a KFERQ like recognition sequence across the lysosomal membrane sequence into the lysosomal lumen. This transport is assisted by cytosolic and lysosomal chaperones as well as LAMP2a. The third pathway, macroautophagy, is currently the best characterised of the three. Macroautophagy employs 34 gene products, so called autophagy related (Atg) proteins, to construct a vesicle, the autophagosome, around its substrate and deliver it for fusion with lysosomes [3]. Autophagosomes are assembled from membranes of the rough endoplasmic reticulum, Golgi apparatus, outer nuclear or mitochondrial membrane, and the cell membrane [4-11]. Autophagosome assembly nucleates around type III phosphatidylinositol (PI) 3 kinase complexes, containing the PI3 kinase hVps34, hVps15, Atg14L and Atg6/Beclin-1 (Figure 1). Elongation of the autophagosome membrane to an isolation membrane is then achieved with the support of two ubiquitin-like systems. In one, Atg12 is activated by the E1-like enzyme Atg7 and then conjugated to Atg5 by the E2-like enzyme Atg10. The resulting conjugate then assembles with Atg16L1 and associates with the isolation membrane to function as an E3-like ligase for the other ubiquitin-like molecule Atg8 [12,13]. In the Atg8 ubiquitin-like conjugation system, Atg8 is first proteolytically processed by Atg4 to remove the five C-terminal amino acids and expose a C-terminal glycine residue for ligation. Then Atg8 is activated by the E1-like enzyme Atg7 and transferred to the E2-like conjugating enzyme Atg3. It gets finally linked to phosphatidylethanolamine in the isolation membrane to mediate extension of the forming autophagosome [14,15]. In addition, one mammalian homologue of Atg8, LC3, has been shown to anchor autophagosome cargo recruitment via p62/SQSTM1, NBR1 and NDP52 [16-19]. Isolation membrane extension leads eventually to the completion of the double-membrane surrounded autophagosome, from whose outer membrane the Atg12 complex with Atg5 and Atg16L1, as well as Atg8 are recycled. Atg8, however, remains attached to the inner autophagosome membrane, and is degraded with the vesicle cargo. Completed autophagosomes can then fuse with late endosomes/multivesicular bodies (MVBs) or lysosomes. These fusion events are also supported or inhibited by PI3 kinase complexes containing Atg6/Beclin-1 and UVRAG or Rubicon instead of Atg14, respectively [20-22]. Fusion with lysosomes requires Rab7 and LAMP2 [23,24], while fusion with MVBs needs Rab11 [25]. In these compartments autophagosome cargo is then degraded by lysosomal hydrolases.

Of the three autophagy pathways, herpesvirus infection and their immune control have been shown to be regulated by macroautophagy. Therefore, we will concentrate in this review primarily on the influence of macroautophagy on herpesvirus infection in vivo, on T cell responses to herpesviruses and on immune escape mechanisms of herpesviruses that target this pathway.

### Restriction of herpesvirus infection by macroautophagy in vivo

Regulation of herpesvirus infection by macroautophagy in vivo has primarily been investigated for herpes simplex virus type 1 (HSV-1), an α-herpesvirus that causes a variety of clinical syndromes ranging from mild mucocutaneous disease to life-threatening viral encephalitis. In part, direct pathogen degradation by macroautophagy, termed "xenophagy", might be involved in this regulation. Early on it was recognized that HSV induces macroautophagy via double-stranded RNA-dependent protein kinase R (PKR), probably after recognizing convergently transcribed double-stranded RNA [26] and through activation of eukaryotic initiation factor (eIF) 2α [27]. While this is probably not the only pathway by which viruses stimulate macroautophagy [28,29], in vivo evidence of the importance of PKR in the restriction of HSV infection in the central nervous system has been provided [30]. Moreover, HSV encodes infected cell protein 34.5 (ICP34.5), an important neurovirulence factor that plays a critical role in the development of fatal encephalitis in both mice and humans [31]. ICP34.5 can counteract PKR mediated HSV restriction and part of its function is blocking macroautophagy [32]. Indeed, recombinant HSV lacking only the macroautophagy inhibiting domain of ICP34.5 is less neurovirulent in mice [33]. HSV that cannot inhibit macroautophagy replicates to lower viral titers and causes less pathology. Again this restriction of ICP34.5 mutant HSV is PKR dependent and does not occur in PKR deficient mice. These data indicate that during cerebral HSV infection macroautophagy would control pathology and viral titers, should the virus not be able to block macroautophagy by its ICP34.5 protein. In addition to this interplay between HSV and macroautophagy during infection of the Central Nervous System (CNS), macroautophagy also affects vaginal HSV infection in mice. Deficiency of macroautophagy in dendritic cells (DCs) due to excision of Atg5 leads to elevated pathology and morbidity of intravaginally infected mice [34]. In combination, these data point towards roles for macroautophagy in the immune control of HSV in neurones as well as a wider systemic role in the priming of immune responses by DCs following intravaginal infection.

Apart from HSV, some in vivo data also exist for a role of macroautophagy inhibition in the growth of Kaposi sarcoma associated herpesvirus (KSHV) infected primary effusion lymphoma (PEL) cells in NOD/SCID mice. KSHV encoded vFLIP, a homolog of cellular FLICE-like inhibitor protein (cFLIP), blocks macroautophagy [35]. However, this inhibition can be compromised by dominant negative fragments of vFLIP, and these even attenuate PEL growth in vivo. Thus, KSHV seems to regulate macroautophagy to promote latently infected B cell growth in vivo.

### Herpesvirus antigen processing for MHC presentation by macroautophagy

As described above, macroautophagy clearly plays an important role in the control of herpesvirus infection in vivo, possibly by orchestrating adaptive immunity in addition to just innately degrading pathogens. Along these lines, macroautophagy has now been shown to influence the adaptive immune response towards herpesviruses. By delivering portions of cytoplasm to lysosomes, macroautophagy provides a route for endogenous proteins to intersect vesicles that form an important part of the MHC class II processing pathway. These proteins are then degraded to generate peptide epitopes which, upon binding into the groove of trafficking MHC class II molecules, are transported to the cell surface for presentation to CD4^+ ^T cells.

Formal demonstration that MHC class II peptide epitopes can be generated by macroautophagy came from studies in murine cells on the antigen processing of the mouse complement C5 protein [36]. The subsequent observation that an MHC class II epitope was processed from a bacterial protein by macroautophagy then clearly showed this pathway was not limited to self-proteins [37]. The first report of an MHC class II epitope being processed from a viral protein by macroautophagy came from studies on a herpesvirus protein. The γ-herpesvirus Epstein Barr virus (EBV) establishes life-long latent infection in MHC class II positive B cells which is maintained by its genome maintenance protein, EBV Nuclear Antigen 1 (EBNA1). Following inhibition of macroautophagy the recognition by EBNA1-specific CD4^+ ^T cells of EBV-transformed B Lymphoblastoid Cell Lines (LCLs), which naturally express physiological levels of EBNA1, was reduced [38]. Interestingly, further work has since shown that ablating EBNA1's nuclear localization sequence (NLS), causing the protein to be present not just in the nucleus but also in the cytoplasm, increases the generation of CD4^+ ^T cell epitopes from EBNA1 by macroautophagy [39]. Restoration of nuclear localization, by the insertion of a heterologous NLS, decreased CD4^+ ^T cell recognition demonstrating that it was indeed sub-cellular localization responsible for this effect. EBNA1, by virtue of its natural localization within the nucleus, enjoys a measure of protection from macroautophagy mediated degradation. Whether other viral nuclear antigens behave similarly remains to be determined.

The studies described above clearly show that macroautophagy is important in the processing of antigens *in vitro*. What then is the relevance of macroautophagy to the adaptive immune response *in vivo*? Mice that have had the essential macroautophagy gene *atg5 *conditionally deleted in their DCs, were less efficient at processing and presenting MHC class II epitopes from endogenous and exogenous antigens leading to impaired Th1 responses, while cross-presentation of exogenous antigens to CD8^+ ^T cells was unaffected [34]. This study clearly demonstrates that macroautophagy plays an important role in the ability of DCs to process and present antigens to CD4^+ ^T cells. However, the normal state of affairs is that viruses infect hosts that have an intact macroautophagy pathway in their immune cells. Given the importance of macroautophagy for the correct functioning of DCs (and therefore adaptive immunity as a whole) it would seem an obvious pathway to be targeted by viruses seeking to subvert immunity. Herpesviruses have evolved a variety of mechanisms to evade host immune responses and targeting autophagy can now be added to their repertoire. HSV-1 expressed ICP34.5 inhibits macroautophagy by binding the cellular protein Atg6/Beclin-1 [33]. Removal of the Beclin-1 binding domain from ICP34.5 did not affect HSV-1 replication in vitro, nor its neurovirulence in *Rag1^-/- ^*mice that lack B and T cells [40]. However, neurovirulence was reduced in immunocompetent mice, a result suggesting that by binding Atg6/Beclin-1 and inhibiting macroautophagy, ICP34.5 interferes with the adaptive immune response to HSV. This was shown to be the case, with mice infected with the mutant virus (unable to inhibit macroautophagy) making much larger HSV-1 specific CD4^+ ^T cell responses relative to mice infected with a control virus [40]. These studies suggest that macroautophagy enhances CD4^+ ^T cell immunity in vivo by assisting viral antigen presentation on MHC class II molecules.

Another study using HSV has revealed that macroautophagy may be important for CD8^+ ^T cell immunity as well. In murine macrophages infected with HSV-1, an MHC class I epitope from the gB protein was processed by the classic proteasomal pathway of antigen presentation during the early stages of infection, but by a vacuolar pathway at later stages [8]. As expected for HSV-1, macroautophagy was inhibited during the early stages of infection. However, at later stages the cells contained not only conventional autophagosomes in the cytoplasm, but also four-layered membrane structures connected to the nucleus; both were positive for LC3, a standard marker of autophagosomes. These unusual multi-membrane autophagosome structures were, like conventional autophagosomes, comprised of membranes from the endoplasmic reticulum and nucleus. The fact that these novel autophagosome structures were not seen in macrophages treated with drugs or stress to induce autophagy raises the possibility that this is a response to virus infection. Interestingly, only conventional autophagosomes were observed in cells infected with an HSV-1 mutant that, through deletion of the ICP34.5 gene, was unable to interfere with conventional macroautophagy. One possibility is that this unusual nuclear macroautophagy is a cellular response to a combination of HSV infection and macroautophagy blockade.

All the work described above has focused on macroautophagy as the mechanism of processing and presentation of viral antigens. It should, however, be noted that in this context other autophagy pathways could also potentially contribute. CMA has been less well studied, but this pathway has also been shown to be capable of generating CD4^+ ^T cell epitopes from cellular proteins [41]. What about viral proteins in infected cells? Unlike macroautophagy, which is generally considered to be sequence unspecific, proteins degraded by CMA require a pentapeptide motif (KFERQ or a biochemically related sequence). Although this may at first seem to compromise CMA's value as an antigen processing pathway, it is important to note that KFERQ-like motifs occur frequently in proteins; for example, almost 30% of cellular proteins contain such a motif [42]. The chances that viruses lack a suitable motif in any of their proteins seem remote and it is likely only to be a matter of time before a virus epitope is shown to be generated by CMA. It will be interesting to see if any of the herpesviruses encode proteins that are substrates of CMA and, if so, whether they have also evolved countermeasures to this processing pathway.

Therefore, intracellular antigen processing for MHC class II presentation via autophagy has now been documented for macroautophagy and CMA. It, however, remains to be explored how autophagy can contribute to extracellular antigen processing and antigen packaging for cross-presentation [43].

### Immune escape from autophagy by herpesviruses

Autophagy has recently been identified as an important intracellular effector mechanism of host immunity. By delivering intracellular bacteria and viruses to lysosomes for destruction via "xenophagy", and by importing viral nucleic acids to immune sensors in the endo/lysosomal compartment, macroautophagy plays a critical role in the defense against a number of chronic intracellular pathogens [1,2,44]. To ensure their own replication and to enhance their life cycle, many microbes have developed countermeasures and escape mechanisms to evade, subvert, or exploit macroautophagy, thereby adding to the cat-and-mouse game of microbial pathogenesis and host-pathogen interactions.

Given the intimate virus-host relationship required to establish a latent state of lifetime persistence in the infected host, it is not surprising that herpesviruses belong to the continuously growing list of viruses that have developed strategies to defeat the antiviral effects of macroautophagy [45-47]. In fact, representatives of all three herpesvirus subfamilies encode proteins or induce cellular signaling pathways that affect macroautophagy.

The best studied example is HSV-1 [48]. A major breakthrough in understanding HSV-1 neurovirulence was the identification of ICP34.5 as an important neurovirulence factor that suppresses macroautophagy in infected cells via two different pathways: the dephosphorylation of translation initiation factor eIF2α and the binding to the macroautophagy-promoting protein Atg6/Beclin-1 [27,33]. The double-stranded RNA-dependent kinase PKR stimulates macroautophagy induction through eIF2α phosphorylation and ICP34.5 is able to antagonize this response by recruiting the host phosphatase PP1α to dephosphorylate eIF2α. In addition, ICP34.5 can also bind to Atg6/Beclin-1 and might prevent it from entering the PI3 kinase complex to induce macroautophagy [49]. While the precise mechanisms by which macroautophagy protects against viral neuropathology are still unclear, it seems likely that macroautophagy functions to restrict viral replication. In ultrastructural studies, virions of the ICP34.5 deletion mutant have been detected within autophagosomes [31,50]. Moreover, metabolic labeling of viral proteins demonstrated increased rates of degradation in the ICP34.5 deletion mutant virus-infected cells compared with wild-type HSV-1 virus-infected cells [50]. These results indicate that virion targeting for xenophagic degradation is compromised by ICP34.5 [33].

Interestingly, other viruses that cause CNS disease also appear to suppress host macroautophagy. Human cytomegalovirus (HCMV), which belongs to the β-herpesvirus family and which causes severe CNS infections in neonates and immunocompromised adults, inhibits host macroautophagy, albeit with mechanisms different from those of HSV-1 [51]. In primary human fibroblasts, HCMV can block macroautophagy by activating the mTOR signaling pathway [52], which is known to suppress macroautophagy. Moreover, lithium chloride-induced macroautophagy, which is mTOR-independent, is also suppressed by HCMV infection [53]. Thus, HCMV is able to inhibit macroautophagy via both mTOR-dependent and -independent pathways [51].

It is worth noting that in addition to these neurotropic viruses, other viruses, such as the oncogenic γ-herpesviruses that infect extraneural tissues, also encode inhibitors of Atg6/Beclin-1 and macroautophagy. Although the role of macroautophagy in γ-herpesvirus pathogenesis is not yet clear, these observations suggest that viral evasion of macroautophagy is probably not restricted to viruses that specifically infect the CNS. All members of the γ-herpesvirus family have in common that they express orthologs of Bcl-2, the cellular prototype inhibitor of apoptosis. These include orf16 of KSHV, herpesvirus saimiri and rhesus rhadinovirus, the BHRF1 and BALF-1 proteins of EBV, and the M11 protein of MHV68 [54]. In this context it is important to mention that apoptosis and macroautophagy are tightly interconnected and coordinately regulated. Besides preventing apoptosis, Bcl-2 also inhibits stress-induced macroautophagic cell death by binding to Atg6/Beclin-1 [55]. Several of the viral homologues of Bcl-2 have been shown to bind to Atg6/Beclin-1 and to prevent it from binding to PI3 kinase complexes and, hence, suppress the initiation of macroautophagy [22,56,57]. Thus, vBcl-2 s of γ-herpesviruses potentially inhibit both apoptosis and macroautophagy by binding to specific effectors of these pathways. This propensity of the γ-herpesvirus vBcl-2 proteins implies that macroautophagy might at least in part impact on viral persistence. Of note, it has recently been reported that maintenance of persistent MHV68 infection is impaired in a mutant virus lacking the anti-macroautophagic activity of vBcl-2 compared to the wild-type virus [56], suggesting that MHV68, via vBcl-2, evades macroautophagy-mediated antiviral responses.

Besides targeting Atg6/Beclin-1, several herpesviruses including KSHV and herpesvirus saimiri can also suppress macroautophagy by expressing vFLIP, a homolog of cFLIP [29]. vFLIP, similarly to cFLIP, can bind to and prevent Atg3 from binding and processing LC3. This enzymatic activity of Atg3 is critical for the lipidation of Atg8/LC3 and the formation of autophagic vacuoles [58].

As mentioned above, EBV codes for two vBcl2 proteins (BALF1 and BHRF1) that are both expressed early during the lytic cycle, implying a protective role of these gene products during viral replication. In addition, both proteins are expressed early after infection of primary B cells [59], and BHRF1 also during the latent phase of infection [60]. Although it is currently not known whether these two proteins bind to Atg6/Beclin-1, these findings might indicate that EBV modulates macroautophagy at least during two phases of its life cycle.

Studies in other viral systems have demonstrated that virus replication, biogenesis, and egress can benefit from an increase in macroautophagy activity [61], and several herpesviruses including EBV, KSHV and varicella-zoster virus (VZV) have recently been demonstrated to not just suppress, but also induce macroautophagy. How VZV induces macroautophagy is unclear, but it does not seem to require late gene products, since the suppression of viral late gene expression with phosphonoacetic acid does not abolish VZV-induced macroautophagy [62]. The EBV latent membrane protein 1 (LMP-1) can induce macroautophagy in a dose-dependent manner [63]. Interestingly, the expression level of EBV LMP-1 also seems to be regulated by macroautophagy, as its level increases if macroautophagy is suppressed by short hairpin RNA (shRNA) targeting Atg6/Beclin-1 or Atg7 [29,63]. Furthermore, RTA, an essential viral protein for KSHV lytic reactivation, enhances macroautophagy and thereby facilitates lytic replication [64].

Thus, herpesviruses can affect macroautophagy either positively or negatively, depending on the virus, the virus' life cycle, and potentially the cellular context. However, with the exception of HSV-1, which suppresses macroautophagy to enhance its replication and pathogenesis, the role of macroautophagy in the life cycle of other herpesviruses still needs to be further elucidated.

## Conclusions

Herpesviruses as one of the most successful families of pathogens have developed mechanisms to inhibit both innate and adaptive immune responses that rely on macroautophagy, and require this regulation for pathogenicity in vivo. These immune escape mechanisms of herpesviruses might, however, also affect functions that macroautophagy has beyond resistance to pathogens. Along these lines they could interfere with clearance of protein aggregates by macroautophagy during CNS infection [65,66], compromise the role of macroautophagy in maintaining genome integrity as a protective mechanism against tumors [67] and impair protective mechanisms of macroautophagy against aging [68]. Thus, the pathomechanisms of chronic herpesvirus infections could potentially extend beyond direct transformation of cells, tissue destruction and immunopathology after lytic reactivation. As the list of herpesvirus associated diseases in humans grows, the potential for novel mechanisms of pathogenesis involving autophagy should be borne in mind.

## Competing interests

The authors declare that they have no competing interests.

## Authors' contributions

GST, JM and CM wrote the manuscript. All authors read and approved the final paper.

## References

[B1] Münz C Enhancing immunity through autophagy Annu Rev Immunol 2009 27 423 429 19105657 10.1146/annurev.immunol.021908.132537

[B2] Mizushima N Levine B Cuervo AM Klionsky DJ Autophagy fights disease through cellular self-digestion Nature 2008 451 1069 1075 10.1038/nature06639 18305538 PMC2670399

[B3] He C Klionsky DJ Regulation mechanisms and signaling pathways of autophagy Annu Rev Genet 2009 43 67 93 10.1146/annurev-genet-102808-114910 19653858 PMC2831538

[B4] Hayashi-Nishino M Fujita N Noda T Yamaguchi A Yoshimori T Yamamoto A A subdomain of the endoplasmic reticulum forms a cradle for autophagosome formation Nat Cell Biol 2009 11 1433 1437 10.1038/ncb1991 19898463

[B5] Yla-Anttila P Vihinen H Jokitalo E Eskelinen EL 3 D tomography reveals connections between the phagophore and endoplasmic reticulum Autophagy 2009 5 1180 1185 10.4161/auto.5.8.10274 19855179

[B6] Yen WL Shintani T Nair U Cao Y Richardson BC Li Z Hughson FM Baba M Klionsky DJ The conserved oligomeric Golgi complex is involved in double-membrane vesicle formation during autophagy J Cell Biol 2010 188 101 114 10.1083/jcb.200904075 20065092 PMC2812853

[B7] Lynch-Day MA Bhandari D Menon S Huang J Cai H Bartholomew CR Brumell JH Ferro-Novick S Klionsky DJ Trs85 directs a Ypt1 GEF, TRAPPIII, to the phagophore to promote autophagy Proc Natl Acad Sci USA 2010 107 7811 7816 10.1073/pnas.1000063107 20375281 PMC2867920

[B8] English L Chemali M Duron J Rondeau C Laplante A Gingras D Alexander D Leib D Norbury C Lippe R Desjardins M Autophagy enhances the presentation of endogenous viral antigens on MHC class I molecules during HSV-1 infection Nat Immunol 2009 10 480 487 10.1038/ni.1720 19305394 PMC3885169

[B9] Hailey DW Rambold AS Satpute-Krishnan P Mitra K Sougrat R Kim PK Lippincott-Schwartz J Mitochondria supply membranes for autophagosome biogenesis during starvation Cell 2010 141 656 667 10.1016/j.cell.2010.04.009 20478256 PMC3059894

[B10] He C Song H Yorimitsu T Monastyrska I Yen WL Legakis JE Klionsky DJ Recruitment of Atg9 to the preautophagosomal structure by Atg11 is essential for selective autophagy in budding yeast J Cell Biol 2006 175 925 935 10.1083/jcb.200606084 17178909 PMC2064702

[B11] Ravikumar B Moreau K Jahreiss L Puri C Rubinsztein DC Plasma membrane contributes to the formation of pre-autophagosomal structures Nat Cell Biol 2010 12 747 757 10.1038/ncb2078 20639872 PMC2923063

[B12] Fujita N Itoh T Omori H Fukuda M Noda T Yoshimori T The Atg16L Complex Specifies the Site of LC3 Lipidation for Membrane Biogenesis in Autophagy Mol Biol Cell 2008 19 2092 2100 10.1091/mbc.E07-12-1257 18321988 PMC2366860

[B13] Hanada T Noda NN Satomi Y Ichimura Y Fujioka Y Takao T Inagaki F Ohsumi Y The Atg12-Atg5 conjugate has a novel E3-like activity for protein lipidation in autophagy J Biol Chem 2007 282 37298 37302 10.1074/jbc.C700195200 17986448

[B14] Nakatogawa H Ichimura Y Ohsumi Y Atg8, a ubiquitin-like protein required for autophagosome formation, mediates membrane tethering and hemifusion Cell 2007 130 165 178 10.1016/j.cell.2007.05.021 17632063

[B15] Xie Z Nair U Klionsky DJ Atg8 Controls Phagophore Expansion during Autophagosome Formation Mol Biol Cell 2008 19 3290 3298 10.1091/mbc.E07-12-1292 18508918 PMC2488302

[B16] Bjorkoy G Lamark T Brech A Outzen H Perander M Overvatn A Stenmark H Johansen T p62/SQSTM1 forms protein aggregates degraded by autophagy and has a protective effect on huntingtin-induced cell death J Cell Biol 2005 171 603 614 10.1083/jcb.200507002 16286508 PMC2171557

[B17] Kirkin V Lamark T Sou YS Bjorkoy G Nunn JL Bruun JA Shvets E McEwan DG Clausen TH Wild P A role for NBR1 in autophagosomal degradation of ubiquitinated substrates Mol Cell 2009 33 505 516 10.1016/j.molcel.2009.01.020 19250911

[B18] Thurston TL Ryzhakov G Bloor S von Muhlinen N Randow F The TBK1 adaptor and autophagy receptor NDP52 restricts the proliferation of ubiquitin-coated bacteria Nat Immunol 2009 10 1215 1221 10.1038/ni.1800 19820708

[B19] Ponpuak M Davis AS Roberts EA Delgado MA Dinkins C Zhao Z Virgin HW Iv Kyei GB Johansen T Vergne I Deretic V Delivery of cytosolic components by p62 endows autophagosomes with unique anti-microbial properties Immunity 2010 32 329 341 10.1016/j.immuni.2010.02.009 20206555 PMC2846977

[B20] Matsunaga K Saitoh T Tabata K Omori H Satoh T Kurotori N Maejima I Shirahama-Noda K Ichimura T Isobe T Two Beclin 1-binding proteins, Atg14L and Rubicon, reciprocally regulate autophagy at different stages Nat Cell Biol 2009 11 385 396 10.1038/ncb1846 19270696

[B21] Zhong Y Wang QJ Li X Yan Y Backer JM Chait BT Heintz N Yue Z Distinct regulation of autophagic activity by Atg14L and Rubicon associated with Beclin 1-phosphatidylinositol-3-kinase complex Nat Cell Biol 2009 11 468 476 10.1038/ncb1854 19270693 PMC2664389

[B22] Liang C Lee JS Inn KS Gack MU Li Q Roberts EA Vergne I Deretic V Feng P Akazawa C Jung JU Beclin1-binding UVRAG targets the class C Vps complex to coordinate autophagosome maturation and endocytic trafficking Nat Cell Biol 2008 10 776 787 10.1038/ncb1740 18552835 PMC2878716

[B23] Jager S Bucci C Tanida I Ueno T Kominami E Saftig P Eskelinen EL Role for Rab7 in maturation of late autophagic vacuoles J Cell Sci 2004 117 4837 4848 10.1242/jcs.01370 15340014

[B24] Tanaka Y Guhde G Suter A Eskelinen EL Hartmann D Lullmann-Rauch R Janssen PM Blanz J von Figura K Saftig P Accumulation of autophagic vacuoles and cardiomyopathy in LAMP-2-deficient mice Nature 2000 406 902 906 10.1038/35022595 10972293

[B25] Fader CM Sanchez D Furlan M Colombo MI Induction of autophagy promotes fusion of multivesicular bodies with autophagic vacuoles in k562 cells Traffic 2008 9 230 250 10.1111/j.1600-0854.2007.00677.x 17999726

[B26] Jacquemont B Roizman B RNA synthesis in cells infected with herpes simplex virus. X. Properties of viral symmetric transcripts and of double-stranded RNA prepared from them J Virol 1975 15 707 713 163916 10.1128/jvi.15.4.707-713.1975 PMC354512

[B27] Talloczy Z Jiang W Virgin HWt Leib DA Scheuner D Kaufman RJ Eskelinen EL Levine B Regulation of starvation- and virus-induced autophagy by the eIF2alpha kinase signaling pathway Proc Natl Acad Sci USA 2002 99 190 195 10.1073/pnas.012485299 11756670 PMC117537

[B28] Delgado MA Elmaoued RA Davis AS Kyei G Deretic V Toll-like receptors control autophagy Embo J 2008 27 1110 1121 10.1038/emboj.2008.31 18337753 PMC2323261

[B29] Lee DY Lee J Sugden B The unfolded protein response and autophagy: herpesviruses rule! J Virol 2009 83 1168 1172 10.1128/JVI.01358-08 18787009 PMC2620921

[B30] Leib DA Machalek MA Williams BR Silverman RH Virgin HW Specific phenotypic restoration of an attenuated virus by knockout of a host resistance gene Proc Natl Acad Sci USA 2000 97 6097 6101 10.1073/pnas.100415697 10801979 PMC18564

[B31] Orvedahl A Levine B Autophagy and viral neurovirulence Cell Microbiol 2008 10 1747 1756 10.1111/j.1462-5822.2008.01175.x 18503639 PMC2737270

[B32] Alexander DE Ward SL Mizushima N Levine B Leib DA Analysis of the role of autophagy in replication of herpes simplex virus in cell culture J Virol 2007 81 12128 12134 10.1128/JVI.01356-07 17855538 PMC2169004

[B33] Orvedahl A Alexander D Talloczy Z Sun Q Wei Y Zhang W Burns D Leib D Levine B HSV-1 ICP34.5 confers neurovirulence by targeting the Beclin 1 autophagy protein Cell Host & Microbe 2007 1 23 35 10.1016/j.chom.2006.12.001 18005679

[B34] Lee HK Mattei LM Steinberg BE Alberts P Lee YH Chervonsky A Mizushima N Grinstein S Iwasaki A In vivo requirement for Atg5 in antigen presentation by dendritic cells Immunity 2010 32 227 239 10.1016/j.immuni.2009.12.006 20171125 PMC2996467

[B35] Lee JS Li Q Lee JY Lee SH Jeong JH Lee HR Chang H Zhou FC Gao SJ Liang C Jung JU FLIP-mediated autophagy regulation in cell death control Nat Cell Biol 2009 11 1355 1362 10.1038/ncb1980 19838173 PMC2802862

[B36] Brazil MI Weiss S Stockinger B Excessive degradation of intracellular protein in macrophages prevents presentation in the context of major histocompatibility complex class II molecules Eur J Immunol 1997 27 1506 1514 10.1002/eji.1830270629 9209504

[B37] Nimmerjahn F Milosevic S Behrends U Jaffee EM Pardoll DM Bornkamm GW Mautner J Major histocompatibility complex class II-restricted presentation of a cytosolic antigen by autophagy Eur J Immunol 2003 33 1250 1259 10.1002/eji.200323730 12731050

[B38] Paludan C Schmid D Landthaler M Vockerodt M Kube D Tuschl T Münz C Endogenous MHC class II processing of a viral nuclear antigen after autophagy Science 2005 307 593 596 10.1126/science.1104904 15591165

[B39] Leung CS Haigh TA Mackay LK Rickinson AB Taylor GS Nuclear location of an endogenously expressed antigen, EBNA1, restricts access to macroautophagy and the range of CD4 epitope display Proc Natl Acad Sci USA 2010 107 2165 2170 10.1073/pnas.0909448107 20133861 PMC2836662

[B40] Leib DA Alexander DE Cox D Yin J Ferguson TA Interaction of ICP34.5 with Beclin 1 modulates herpes simplex virus type 1 pathogenesis through control of CD4+ T-cell responses J Virol 2009 83 12164 12171 10.1128/JVI.01676-09 19759141 PMC2786728

[B41] Zhou D Li P Lott JM Hislop A Canaday DH Brutkiewicz RR Blum JS Lamp-2a facilitates MHC class II presentation of cytoplasmic antigens Immunity 2005 22 571 581 10.1016/j.immuni.2005.03.009 15894275

[B42] Chiang HL Dice JF Peptide sequences that target proteins for enhanced degradation during serum withdrawal J Biol Chem 1988 263 6797 6805 3360807

[B43] Münz C Antigen processing via autophagy--not only for MHC class II presentation anymore? Curr Opin Immunol 2010 22 89 93 20149615 10.1016/j.coi.2010.01.016 PMC3082731

[B44] Levine B Kroemer G Autophagy in the pathogenesis of disease Cell 2008 132 27 42 10.1016/j.cell.2007.12.018 18191218 PMC2696814

[B45] Lin LT Dawson PW Richardson CD Viral interactions with macroautophagy: a double-edged sword Virology 2010 402 1 10 10.1016/j.virol.2010.03.026 20413139 PMC7111941

[B46] Orvedahl A Levine B Viral evasion of autophagy Autophagy 2008 4 280 285 18059171 10.4161/auto.5289 PMC3508671

[B47] Sir D Ou JH Autophagy in viral replication and pathogenesis Mol Cells 2010 29 1 7 10.1007/s10059-010-0014-2 20077024 PMC3115743

[B48] Koelle DM Corey L Herpes simplex: insights on pathogenesis and possible vaccines Annu Rev Med 2008 59 381 395 10.1146/annurev.med.59.061606.095540 18186706

[B49] He C Levine B The Beclin 1 interactome Curr Opin Cell Biol 2010 22 140 149 10.1016/j.ceb.2010.01.001 20097051 PMC2854269

[B50] Talloczy Z Virgin HWt Levine B PKR-dependent autophagic degradation of herpes simplex virus type 1 Autophagy 2006 2 24 29 16874088 10.4161/auto.2176

[B51] Chaumorcel M Souquere S Pierron G Codogno P Esclatine A Human cytomegalovirus controls a new autophagy-dependent cellular antiviral defense mechanism Autophagy 2008 4 46 53 18340111 10.4161/auto.5184

[B52] Jung CH Ro SH Cao J Otto NM Kim DH mTOR regulation of autophagy FEBS Lett 2010 584 1287 1295 10.1016/j.febslet.2010.01.017 20083114 PMC2846630

[B53] Sarkar S Floto RA Berger Z Imarisio S Cordenier A Pasco M Cook LJ Rubinsztein DC Lithium induces autophagy by inhibiting inositol monophosphatase J Cell Biol 2005 170 1101 1111 10.1083/jcb.200504035 16186256 PMC2171537

[B54] Polster BM Pevsner J Hardwick JM Viral Bcl-2 homologs and their role in virus replication and associated diseases Biochim Biophys Acta 2004 1644 211 227 10.1016/j.bbamcr.2003.11.001 14996505

[B55] Pattingre S Tassa A Qu X Garuti R Liang XH Mizushima N Packer M Schneider MD Levine B Bcl-2 antiapoptotic proteins inhibit Beclin 1-dependent autophagy Cell 2005 122 927 939 10.1016/j.cell.2005.07.002 16179260

[B56] E X Hwang S Oh S Lee JS Jeong JH Gwack Y Kowalik TF Sun R Jung JU Liang C Viral Bcl-2-mediated evasion of autophagy aids chronic infection of gammaherpesvirus 68 PLoS Pathog 2009 5 e1000609 10.1371/journal.ppat.1000609 19816569 PMC2752191

[B57] Oh S E X Hwang S Liang C Autophagy evasion in herpesviral latency Autophagy 2010 6 151 152 10.4161/auto.6.1.10536 19923915 PMC2847627

[B58] Tanida I Ueno T Kominami E LC3 conjugation system in mammalian autophagy Int J Biochem Cell Biol 2004 36 2503 2518 10.1016/j.biocel.2004.05.009 15325588 PMC7129593

[B59] Altmann M Hammerschmidt W Epstein-Barr virus provides a new paradigm: a requirement for the immediate inhibition of apoptosis PLoS Biol 2005 3 e404 10.1371/journal.pbio.0030404 16277553 PMC1283332

[B60] Kelly GL Long HM Stylianou J Thomas WA Leese A Bell AI Bornkamm GW Mautner J Rickinson AB Rowe M An Epstein-Barr virus anti-apoptotic protein constitutively expressed in transformed cells and implicated in burkitt lymphomagenesis: the Wp/BHRF1 link PLoS Pathog 2009 5 e1000341 10.1371/journal.ppat.1000341 19283066 PMC2652661

[B61] Dreux M Chisari FV Viruses and the autophagy machinery Cell Cycle 2010 in press 20305376 10.4161/cc.9.7.11109

[B62] Takahashi MN Jackson W Laird DT Culp TD Grose C Haynes JI Benetti L Varicella-zoster virus infection induces autophagy in both cultured cells and human skin vesicles J Virol 2009 83 5466 5476 10.1128/JVI.02670-08 19297471 PMC2681990

[B63] Lee DY Sugden B The latent membrane protein 1 oncogene modifies B-cell physiology by regulating autophagy Oncogene 2008 27 2833 2842 10.1038/sj.onc.1210946 18037963

[B64] Wen HJ Yang Z Zhou Y Wood C Enhancement of autophagy during lytic replication by the Kaposi's sarcoma-associated herpesvirus replication and transcription activator J Virol 2010 84 7448 7458 10.1128/JVI.00024-10 20484505 PMC2897602

[B65] Hara T Nakamura K Matsui M Yamamoto A Nakahara Y Suzuki-Migishima R Yokoyama M Mishima K Saito I Okano H Mizushima N Suppression of basal autophagy in neural cells causes neurodegenerative disease in mice Nature 2006 441 885 889 10.1038/nature04724 16625204

[B66] Komatsu M Waguri S Chiba T Murata S Iwata J Tanida I Ueno T Koike M Uchiyama Y Kominami E Tanaka K Loss of autophagy in the central nervous system causes neurodegeneration in mice Nature 2006 441 880 884 10.1038/nature04723 16625205

[B67] Mathew R Karp CM Beaudoin B Vuong N Chen G Chen HY Bray K Reddy A Bhanot G Gelinas C Autophagy suppresses tumorigenesis through elimination of p62 Cell 2009 137 1062 1075 10.1016/j.cell.2009.03.048 19524509 PMC2802318

[B68] Zhang C Cuervo AM Restoration of chaperone-mediated autophagy in aging liver improves cellular maintenance and hepatic function Nat Med 2008 14 959 965 10.1038/nm.1851 18690243 PMC2722716

